# Molecular tools for identification of shark species involved in depredation incidents in Western Australian fisheries

**DOI:** 10.1371/journal.pone.0210500

**Published:** 2019-01-11

**Authors:** Seema Fotedar, Sherralee Lukehurst, Gary Jackson, Michael Snow

**Affiliations:** 1 Department of Primary Industries and Regional Development, Government of Western Australia, Hillarys, Western Australia, Australia; 2 Centre for Evolutionary Biology, School of Biological Sciences, The University of Western Australia, Crawley, Western Australia, Australia; Department of Agriculture and Water Resources, AUSTRALIA

## Abstract

Shark depredation is an issue of concern in some Western Australian recreational and commercial fisheries where it can have economic, social and ecological consequences. Knowledge of the shark species involved is fundamental to developing effective management strategies to mitigate the impacts of depredation. Identification of the species responsible is difficult as direct observation of depredation events is uncommon and evaluating bite marks on fish has a high degree of uncertainty. The use of trace DNA techniques has provided an alternative method for species identification. We demonstrate proof of concept for a targeted DNA barcoding approach to identify shark species using trace DNA found at bite marks on recovered remains of hooked fish. Following laboratory validation, forensic analysis of swabs collected from samples of bitten demersal fish, led to the definitive identification of shark species involved in 100% of the incidences of depredation (n = 16).

## Introduction

Depredation in a fisheries context is the partial or complete removal of fish from fishing gear by non-target species. While a range of taxa have been observed/implicated in depredation events including cetaceans [1–3], pinnipeds [4], squids [5], and large teleosts (e.g. cods), by far the most common taxa involved are sharks [6–8].

Depredation is an ecological and economic issue worldwide, occurring in commercial and recreational fisheries [9, 10]. In north-western Australia, anecdotal observations from recreational, charter and commercial fishers suggest that depredation by sharks may have increased in recent years leading to the assumption by some of a general increase in shark abundance. The case to allow increased commercial shark catches as a solution to reduce depredation has been made in Western Australia [11]. However, there is no direct evidence to support this assumption as these observations might equally reflect changes in distribution and behaviour of a variety of shark species.

Natural resource managers are currently faced with a lack of adequate information on the actual level of interactions and the various shark species involved. Fundamental to the development of any effective depredation management strategy is an improved understanding of the depredating species. This is likely to vary across the distribution of recreational and commercial fisheries which extend throughout the temperate and tropical geographical regions of Western Australia.

Accurate identification of the species involved is problematic, since depredation events are rarely directly observed and many species can only be distinguished by subtle differences in morphological characteristics. Identification of the depredating species is often based on visual identification of sharks caught in the process of consuming hooked fish [10], stomach content analysis of sharks caught in the area [12] and interpretation of evidence such as bite characteristics and teeth marks found on the partially consumed prey. However, the accuracy and consistency of this approach varies based on the expertise of the observer [13].

DNA barcoding is the use of a specific mitochondrial DNA (mtDNA) gene region (cytochrome oxidase subunit 1; COI) to recognize animal species by comparison with validated reference sequences. However, any gene region (mtDNA or nuclear DNA) can be used provided it is diagnostic for the species under consideration and reference sequences are available [14]. DNA testing provides a rapid and accurate means for assigning a specimen to a species that is particularly useful when the sample lacks sufficient morphological characters for routine taxonomic identification (e.g. with a fish fillet) or if the morphological characters are poorly defined (e.g. pre-caudal vertebral counts in whaler sharks, Carcharhinidae), if no diagnostic morphological characters are known (e.g. cryptic species) or if the state of preservation precludes morphological analysis [14]. An increasing number of published papers have presented results on the application of genetic identification techniques for various shark species, using samples from fins and other body parts [15–22]. DNA testing for species identification has become a tool for the investigation of acts of alleged wildlife crime [23], studying food web interactions [24, 25] and more recently has been used to identify predator species [26–30].

In an effort to better understand the species of sharks involved in fish depredation in a Western Australian commercial line-based fishery, this study was conducted to evaluate the development of a molecular tool for identification of depredator species without any *a priori* observation or other evidence. Here we report on a new method, based on the principles of DNA barcoding, to recover and identify trace DNA left by depredator species at bite locations on hooked fish following depredation events.

## Methods

### Development of databases and alignments

Sequences (max. 5 per species) for the cytochrome oxidase subunit 1 (CO1) and cytochrome B (CytB) gene regions for the shark and teleost species commonly encountered in the West Coast and Gascoyne Coast bioregions of Western Australia were downloaded from GenBank/BOLD databases where available. In addition, tissue samples (muscle or fin clips) from the target shark and teleost species (Table 1) were also obtained from the reference archives of the Department of Primary Industries and Regional Development (DPIRD), Western Australia. Samples of shark species included in these reference archives were collected by experienced scientific staff during long-term fishery-dependent and -independent sampling programs where species identification protocols and staff training were based on identification keys reported by Last and Stevens [31]. These reference samples were also used for development and testing of the diagnostic utility of our species-specific primers.

**Table 1:** Shark and teleost species tested.

Common Name	Species	GenBank accession number	
		CO1	CytB
**Sharks**			
Spinner	*Carcharhinus brevipinna*	MG811816	MG811803
Dusky	*Carcharhinus obscurus*	MG811817	MG811804
Grey reef	*Carcharhinus amblyrhynchos*	MG811818	MG811805
Milk	*Rhizoprionodon acutus*	MG811819	MG811806
Pigeye	*Carcharhinus amboinenis*	MG811820	MG811807
Sliteye	*Loxodon macrorhinus*	MG811821	MG811808
Spot-tail	*Carcharhinus sorrah*	MG811822	MG811809
Tiger	*Galeocerdo cuvier*	MG811823	MG811810
Sandbar	*Carcharhinus plumbeus*	MG811824	MG811811
Zebra	*Stegostoma fasciatum*	MG811825	MG811812
Australian Blacktip	*Carcharhinus tilstoni*	MG811826	MG811813
Blacktip	*Carcharhinus limbatus*	MG811827	MG811814
Grey nurse	*Carcharias taurus*	MG811828	MG811815
**Teleosts**			
Pink snapper	*Pagrus auratus*	MK092067	MK092072
Goldband snapper	*Pristipomoides multidens*	MK092068	MK092073
Red emperor	*Lutjanus sebae*	MK092069	MK092074
Gold spotted rockcod	*Epinephelus coioides*	MK092070	MK092075
Spangled emperor	*Lethrinus nebulosus*	MK092071	MK092076

Total genomic DNA was extracted from samples using the Favorprep Tissue Genomic DNA Extraction Mini Kit according to the manufacturer’s instructions (FavorGen BioTech Corp, Taiwan). Polymerase chain reactions (PCR) targeting mitochondrial CO1 and CytB gene regions were conducted using primer sets FishF1/R1 [32] and GludgL/CB2H [33] respectively. Each PCR reaction contained 5μL of MyTaq Reaction Buffer (Bioline), 0.5 μL of MyTaq DNA Polymerase (5U/ μL, Bioline), 2μL of each primer (2.5 μM), 1.25μL BSA (10 mg/mL, Fisher Biotec) and 2μL of target DNA in a final volume of 25μL. PCR reactions were conducted in an Applied Biosystems (ThermoFisher Scientific) Veriti thermal cycler. Amplification products were visualized on 1.5% agarose (Fisher Biotec) gels stained with GelRed (Biotium) alongside a 100 base pair (bp) molecular weight marker (Axygen Biosciences, California USA), under UV light. Bi-directional sequencing of PCR products was performed using a Sanger sequencing service provided by the Australian Genome Research Facility (AGRF), Perth.

Sequences were trimmed and edited using the Sequencher software package version 5.4.6 (Gene Codes Corporation). Individual species identifications were verified by similarity-based searches on the Barcode of Life Database (BOLD) [34] and the NCBI BLAST database [35]. Sequences for the two gene regions were deposited in NCBI GenBank (Table 1).

### Shark-specific primer design and evaluation

Shark and teleost sequences from this study and additional sequences obtained from GenBank were aligned using Clustal W within the Geneious v. 11.0.2 software package [36] for each gene region, resulting in generation of two aligned datasets for each target group.

**Fig 1:**
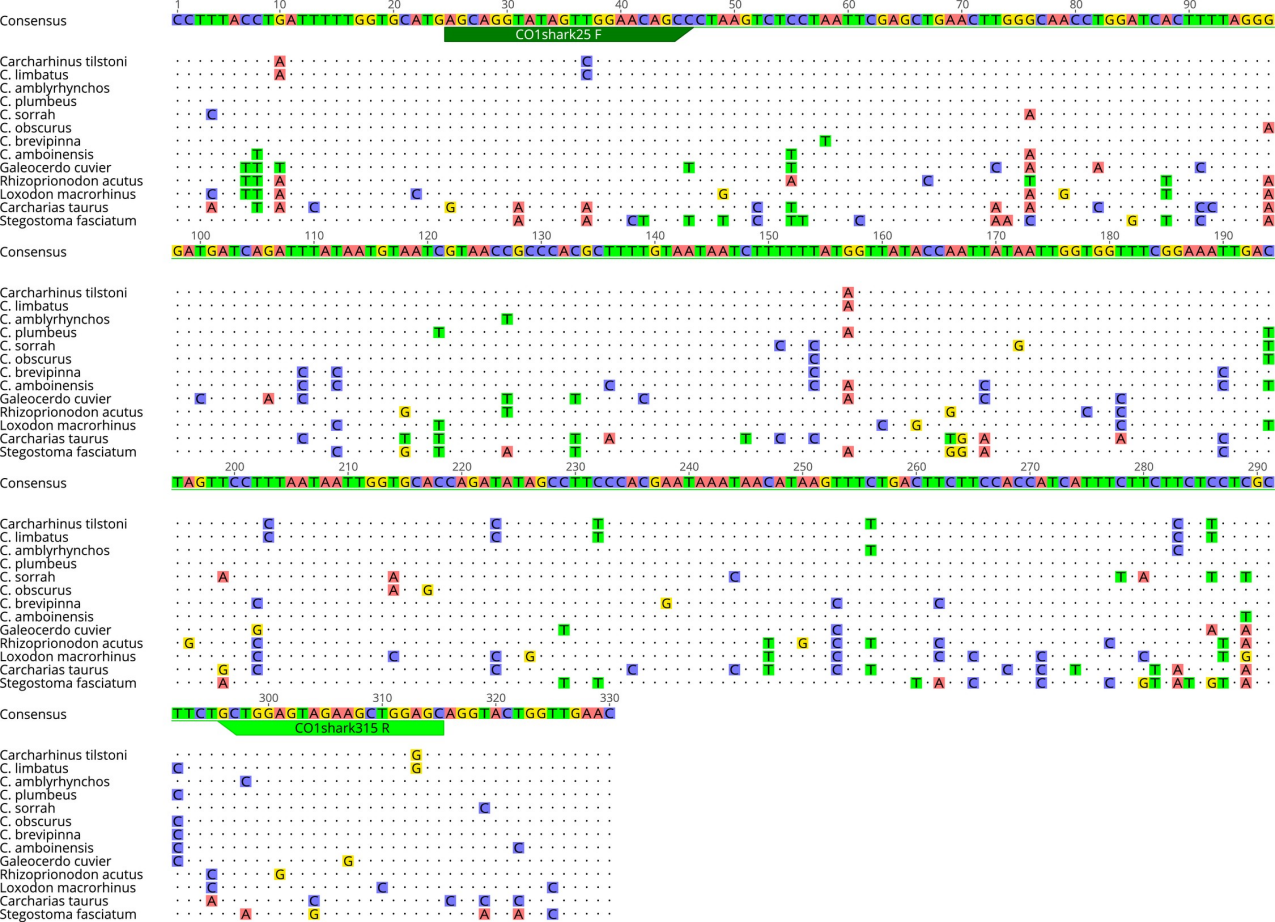
Alignment of shark species CO1 consensus sequences showing position of ShSPs. Nucleotide positions that are identical to the consensus sequence are depicted by dots “.” whereas nucleotide positions that differ from the consensus are depicted with the symbol of the actual nucleotide (A, C, G or T).

**Fig 2:**
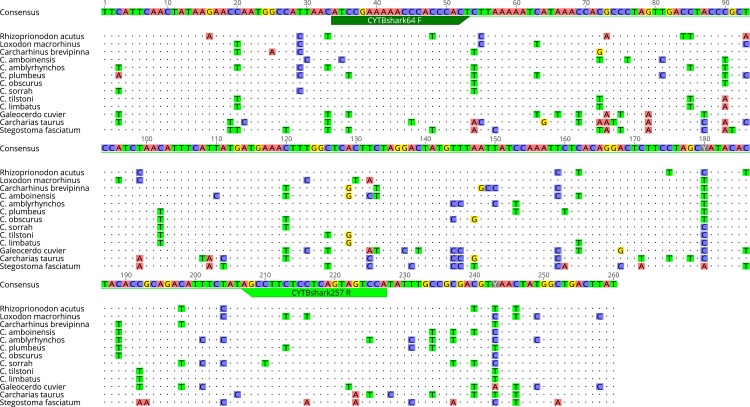
Alignment of shark species CytB consensus sequences showing position of ShSPs. Nucleotide positions that are identical to the consensus sequence are depicted by dots “.” whereas nucleotide positions that differ from the consensus are depicted with the symbol of the actual nucleotide (A, C, G or T).

To avoid the requirement for and problems with blocking primers [37], we elected to design primers that were capable of generic amplification of target shark species (Table 1), but would specifically preclude the amplification of abundant DNA from non-target teleost species. Furthermore, within the selected genome region, sufficient inter-specific variability must exist, such that the level of intra-specific variability does not confound confident species assignment. Two sets of putative shark-specific primers (ShSPs) were designed to fit the discriminatory criteria above, using the Geneious (Biomatters Ltd) primer design software (Table 2). Annealing sites for both sets of ShSPs were positioned internally to the forward and reverse universal CO1 (Fig 1) and CytB (Fig 2) primers. Short fragment lengths were targeted to improve amplification of degraded and trace DNA.

**Table 2:** Shark specific primers (ShSPs) designed, along with their sequences and expected amplicon sizes.

Primer name	Primer sequence	Product size (bp)
CO1shark25F	5'AGCAGGTATAGTTGGAACAGCCC3'	248
CO1shark315R	5'GCTCCAGCTTCTACTCCAGC3'	
CYTBshark64F	5'ATCCGAAAAACCCACCCACT3'	153
CYTBshark257R	5'TGGACTACTGAGGAGAAGGCT3'	

Putative ShSPs were initially evaluated against two reference shark and two teleost species using a Veriti thermal cycler (Applied Biosystems). The reactions were optimised using gradient temperature tests, resulting in the following selected conditions: an initial denaturation at 95°C for 3 min, followed by 35 cycles at 95°C for 30s, 64°C for 45s, 72°C for 1 min and a final extension step of 72°C for 5 min. The shark-specific primers were further tested against the remaining reference shark (n = 11) and teleost (n = 3) samples for their utility in discriminating the two groups.

To determine the specificity of the primers in samples with low amounts of target template, PCRs were conducted on a ten-fold dilution series of DNA extracted from shark tissue (n = 3, starting concentration approx. 20ng/μL). Equal amounts of shark DNA (n = 3, from the tenfold dilution series) and teleost DNA (n = 5, concentration approx. 3 ng/μL) template were mixed, amplified and PCR products sequenced to verify the discriminatory ability of the ShSPs when shark DNA was in low abundance compared to teleost species DNA.

### DNA extraction from swabs

To determine the most suitable DNA extraction method, a preliminary test was carried out. Multiple sterile swabs (COPAN FLOQSwabs, COPAN Flock Technologies) were used to swab the muscle tissue of a pink snapper (*Pagrus auratus*) and were carefully returned to their sterile storage tubes and stored at -20°C until required. The tip of each swab was cut and placed in a separate 2 mL microcentrifuge tube. DNA was extracted using three different commercially available extraction methods: Qiagen QIAmp Stool Kit (n = 4), Qiagen DNeasy Blood & Tissue DNA extraction kit (n = 4) and Favorgen FavorPrep stool kit (n = 4), following the manufacturer’s instructions, with a final elution volume of 50μL.

### Screening incidences of depredation

Samples were obtained during commercial line-based fishing activity conducted in the Gascoyne Coast bioregion of Western Australia. Following the visual identification of significant evidence of depredation in retrieved fish, the captured teleost species were identified and sterile swabs were used to swab the bite marks. Swabs were carefully returned to their sterile storage tube and frozen for transfer to the laboratory and subsequent analysis. Swabs were obtained from 16 incidents of depredation for initial method development and demonstration of feasibility of the approach.

Swabs were stored in the freezer until extraction. DNA was extracted using the QIAmp Stool Kit, following manufacturer’s instructions, with a final elution volume of 50 μL. The two primer sets (Table 2) designed in this study were utilized for screening swab samples using the PCR cycling conditions detailed above. Each PCR reaction contained 5μL of MyTaq Reaction Buffer (Bioline), 0.5 μL of MyTaq DNA Polymerase (5U/ μL, Bioline), 2μL of each primer (2.5 μM), 1.25μL BSA (10 mg/mL, Fisher Biotec), 6–10μL of gDNA and water in a final volume of 25μL. A range of both negative and positive controls (Table 3) were included to ensure assay integrity and to exclude the likelihood of contamination introduced during the sampling and laboratory handling stages. Positive PCR products were sent for commercial Sanger sequencing (AGRF, Perth) and individual sequences determined.

**Table 3:** Assay controls included in each experiment and their purposes are outlined.

Control	Description	Purpose
A	Swab obtained from intact fish caught from the area of depredation incident	Negative control to exclude likelihood of environmental DNA influencing assay
B	Blank swab	Negative control to exclude likelihood of contamination of swab or processing area
C	Swab obtained from working surfaces	Negative control to ensure contamination not introduced during laboratory processing
D	Negative extraction control	Negative control to ensure contamination not introduced during laboratory processing
E	PCR no template control	Negative control to ensure contamination not introduced during laboratory processing
F	Shark DNA	Positive control used to demonstrate efficacy of amplification

Sequences obtained from swabs were compared by similarity-based searches on the BOLD [34] and the NCBI GenBank database [35]. Sequences were also verified by pairwise identity comparison to the shark reference database collection described above, by alignment with Clustal W in Geneious.

## Results

A dataset and associated alignment of CO1 (655bp) and CytB (426bp) sequences were obtained for the target shark and teleost species. Sequences from this study are accessible from the GenBank data base (see Table 1 for accession numbers). A consensus sequence was generated for each of the targeted shark species based on alignment of sequences from this study and those downloaded from GenBank. The CO1 and CytB regions sequenced represented fragments of functional mitochondrial genes and showed no stop codons, insertions, and/ or deletions making these suitable for primer design.

**Fig 3:**
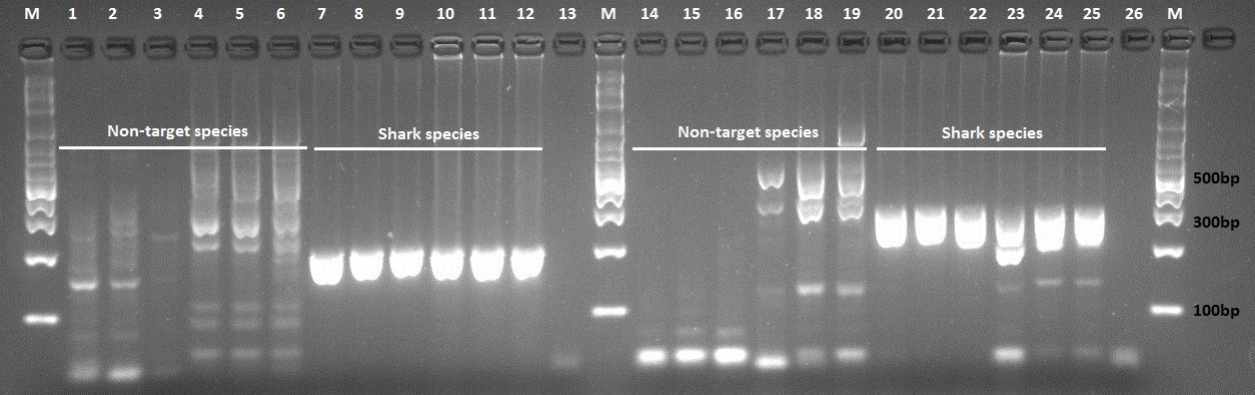
Gel showing improved target specificity with increase in annealing temperature. Amplification reactions using CytB ShSPs (lanes1-13) and CO1 ShSPs (lanes 14–26). Samples: *Pagrus auratus* lanes 1–3 and 14–16; *Lutjanus sebae* lanes 4–6 and 17–19; *Carcharhinus obscurus* lanes 7–9 and 20–22 and *Stegostoma fasciatum* lanes 10–12 and 23–25. Each sample was tested at 60°, 62° and 64°C annealing temperature. Lanes 13 and 26 are no template PCR controls. Lanes labelled M contain the 100bp molecular weight marker (Axygen Biosciences).

**Fig 4:**
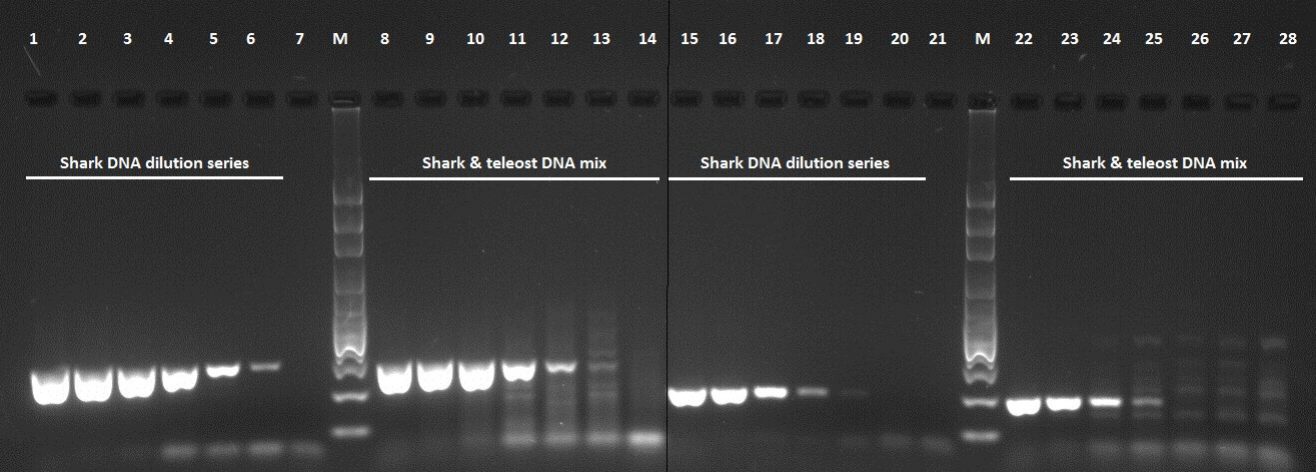
Gel showing examples of amplification of low abundance target DNA in serially diluted and mixed samples, with shark-specific primers (ShSPs). CO1 PCR products in lanes 1–14; CytB PCR products in lanes 15–28. Lanes 7 and 21 are no template PCR reaction controls. Lanes 14 and 28 non-target DNA only controls. Lanes labelled M contain the 100bp molecular weight marker (Axygen Biosciences).

The two newly designed primer sets exhibited specificity to the shark samples tested (n = 13). The teleost (non-target species) samples displayed faint non-specific amplification at lower annealing temperatures which was reduced when annealing temperature was increased to 64°C, examples shown in Fig 3. Both of the ShSPs maintained specificity in reactions containing low abundance DNA. Amplification was successful in CO1 reactions containing as low as 10−^4^ dilution of shark DNA in both the shark only and mixed (shark + teleost DNA) samples whereas CytB amplified only up to 10^−3^ dilution (Fig 4).

Analysis of consensus sequences for target shark species in this study confirmed that a significant inter-specific variability existed, in the region targeted by the ShSPs, to confidently discriminate species in all cases, with the exception of blacktip sharks *C*. *limbatus* and *C*. *tilstoni* (99% nucleotide identity, Table 4). For CytB, this variation was generally above 5%, whereas for CO1, the species discriminatory power was somewhat less. The region targeted by the shark specific CO1 primers included the species-specific diagnostic mutations identified by Ovenden and co-workers [38] for distinguishing the two blacktip species.

**Table 4:** Percentage identity of sequences obtained using ShSPs for CO1 below the diagonal and ShSPs for CytB above the diagonal.

	*Rhizoprionodon acutus*	*Loxodon macrorhinus*	*Carcharhinus brevipinna*	*Carcharhinus amboinensis*	*Carcharhinus tilstoni*	*Carcharhinus limbatus*	*Carcharhinus plumbeus*	*Carcharhinus amblyrhynchos*	*Carcharhinus obscurus*	*Carcharhinus sorrah*	*Galeocerdo cuvier*	*Carcharias taurus*	*Stegostoma fasciatum*
***Rhizoprionodon acutus***		90	87	86	89	90	86	88	87	91	84	82	80
***Loxodon macrorhinus***	90		87	87	89	89	91	89	90	91	82	81	79
***Carcharhinus brevipinna***	90	90		92	90	90	90	90	92	92	82	80	78
***Carcharhinus amboinensis***	88	88	95		90	92	90	88	92	89	82	80	79
***Carcharhinus tilstoni***	90	88	93	93		99	91	90	92	93	83	81	82
***Carcharhinus limbatus***	89	88	94	94	99		91	90	92	93	83	81	81
***Carcharhinus plumbeus***	90	90	95	96	96	97		93	95	92	80	80	78
***Carcharhinus amblyrhynchos***	92	89	95	94	98	97	97		94	93	82	80	81
***Carcharhinus obscurus***	90	90	95	95	95	96	98	96		95	82	84	81
***Carcharhinus sorrah***	88	88	92	94	93	93	94	94	95		85	82	80
***Galeocerdo cuvier***	88	85	91	92	90	91	92	92	90	89		79	80
***Carcharias taurus***	84	83	85	86	85	84	85	85	85	85	85		83
***Stegostoma fasciatum***	84	83	83	85	85	85	85	85	83	83	84	83	

DNA was successfully extracted from the control swabs using the three methods tested with concentrations ranging from 2–18 ng/μL. Swabs extracted with the DNeasy Blood and Tissue kit gave concentrations at the lower end of the range and had poor amplification success. The two stool kits tested gave similar results, however, the QIAmp Stool kit method was determined to be optimal based on ease of protocol, consistency of amplification strength and quality of sequences obtained using the designed ShSPs. Controls performed as expected with no contamination observed.

All 16 incidences of depredation were identified by ShSPs as originating from a range of shark species based on independent but parallel CO1 and CytB analysis (Table 5) Five of the incidences were confidently assigned to *C*. *limbatus*, three each to *C*. *obscurus*, *C*. *plumbeus*, *C*. *amboinensis* and one each to *Rhizoprionodon acutus* and *C*. *taurus* (Table 5). Sequences obtained from swab samples were 100% identical to shark sequences from our reference database suggestive of potentially low levels of intraspecific-diversity for these markers.

**Table 5:** Sequence based identification of shark species involved in incidents of depredation.

Sample number	Teleost swabbed	Blast % (GenBank and BOLD)		Identification
		CO1	CytB	
1	*Lutjanus sebae*	100% *Carcharhinus plumbeus/altimus*	100% *Carcharhinus plumbeus*	*Carcharhinus plumbeus*
2	*Pagrus auratus*	100% *Carcharias taurus*	100% *Carcharias taurus*	*Carcharias taurus*
3	*Pagrus auratus*	100% *Carcharhinus obscurus/ galapagensis*	100% *Carcharhinus obscurus*	*Carcharhinus obscurus*
4	*Pagrus auratus*	100% C. *limbatus/brevipinna/ leiodon/amblyrhynchoides/leucas*	99% *Carcharhinus limbatus*	*Carcharhinus limbatus*
5	*Pagrus auratus*	100% C. *limbatus/brevipinna/ leiodon/amblyrhynchoides/leucas*	99% *Carcharhinus limbatus*	*Carcharhinus limbatus*
6	*Pagrus auratus*	100% C. *limbatus/brevipinna/ leiodon/amblyrhynchoides/leucas*	99% *Carcharhinus limbatus*	*Carcharhinus limbatus*
7	*Pagrus auratus*	100% C. *limbatus/brevipinna/ leiodon/amblyrhynchoides/leucas*	99% *Carcharhinus limbatus*	*Carcharhinus limbatus*
8	*Pagrus auratus*	100% C. *limbatus/brevipinna/ leiodon/amblyrhynchoides/leucas*	99% *Carcharhinus limbatus*	*Carcharhinus limbatus*
9	*Pagrus auratus*	100% *Carcharhinus obscurus/ galapagensis*	100% *Carcharhinus obscurus*	*Carcharhinus obscurus*
10	*Pagrus auratus*	100% *Carcharhinus obscurus/ galapagensis*	100% *Carcharhinus obscurus*	*Carcharhinus obscurus*
11	*Lethrinus nebulosus*	100% *Carcharhinus amboinensis*	100% *Carcharhinus amboinensis*	*Carcharhinus amboinensis*
12	*Lutjanus sebae*	100% *Carcharhinus amboinensis*	100% *Carcharhinus amboinensis*	*Carcharhinus amboinensis*
13	*Lethrinus nebulosus*	100% *Carcharhinus amboinensis*	100% *Carcharhinus amboinensis*	*Carcharhinus amboinensis*
14	*Pristipomoides multidens*	100% *Carcharhinus plumbeus/altimus*	100% *Carcharhinus plumbeus*	*Carcharhinus plumbeus*
15	*Pristipomoides multidens*	100% *Carcharhinus plumbeus/altimus*	100% *Carcharhinus plumbeus*	*Carcharhinus plumbeus*
16	*Pristipomoides multidens*	100% *Rhizoprionodon acutus*	93% *Glyphis fowlerae*	*Rhizoprionodon acutus*

## Discussion

This study details the first known development of a molecular method suited to the identification of species involved in depredation events in commercial line-based fisheries without reliance on direct observation. Trace shark DNA, collected on swabs from bite lacerations on the remains of caught fish, was successfully recovered and sequenced leading to definitive identification of depredator species involved in all 16 sampled incidences of shark depredation. The study, clearly demonstrates the potential for application of this method to better understand the nature and extent of depredating shark species within recreational and commercial fisheries.

The genetic identification of depredators is considered the gold-standard method in wildlife forensic pathology [39] and its efficacy has been demonstrated in both terrestrial [26,27,29,30] and marine animals [28]. Both mitochondrial and nuclear DNA have been used to determine species of processed products [40–42, 17] and to investigate wildlife attacks [27]. The ability to recover mitochondrial DNA from challenging forensic samples such as saliva traces associated with bites is known to be greater than for nuclear genes likely due to mitochondrial DNA being present as multiple copies (up to 15) within each of the multiple mitochondria found within each cell as compared to nuclear genes of which only 2 copies are present in each cell. Furthermore, mitochondria themselves may offer some protection against natural DNA degradation processes due to their encapsulation in a protective protein coat [39] within a cell. The current study, therefore, selected mitochondrial gene markers.

Identification via universally agreed DNA barcodes (e.g. CO1 for animals) is based on the observation that intraspecific divergence is usually lower than interspecific divergence (e.g. barcoding “gap”) [43]. A gap that can confidently differentiate species can be variable depending on the taxa of interest and a between-species cut-off of 2% has been suggested [44, 45]. However, divergence between species can fall below this level for some taxa [32] and can be confounded by a lack of disparity between intra-specific and inter-specific variation [46]. In some cases, the species may be genetically distinguishable on the basis of diagnostic mutations. For example, in the case of blacktip sharks, *C*. *limbatus and C*. *tilstoni*, which are morphologically indistinguishable externally and have been found to hybridise [47–49], the species can be distinguished by two diagnostic mutations in the COI gene [38,21,48,49]. DNA barcoding has been successfully applied to the discrimination of many shark species from Australian waters [17, 21].

In situations where taxa share sequences with less than 1% divergence, multiple species assignments may be made to closely related congeners [17]. Multiple gene markers may need to be assessed for confident species identification. While a number of genetic markers (ITS2, ND4, CytB) have been used in various studies, there are gaps in verified publicly available sequence data. In addition, the accuracy of sequence-based identification depends on the accuracy of the data provided in the online databases and misidentifications have been observed. Therefore, we compiled sequence data for identified samples of shark species commonly encountered in the West Coast and Gascoyne bioregion.

The CO1 and CytB genes contained a relatively high level of inherent inter-species variability making the selected regions well suited to the purpose of species discrimination. Species assignments could be made with a higher degree of confidence based on CytB due to a larger barcoding gap, however, parallel analysis of both CO1 and CytB markers resulted in consistent assignments in all cases.

An important consideration in the design of molecular methods aimed at investigating trophic interactions is a strategy to ensure that the predominance of one species’ DNA (e.g. the target hooked teleost species in the case of a depredation event) within a sample does not bias or restrict the identification of others for which only forensic quantities or degraded DNA may be present (e.g. the species responsible for a depredation event). Such a phenomenon has previously been reported [50, 51] and can be overcome through strategies such as the use of blocking primers [37] or targeted DNA digestion using restriction enzymes [51,52] to ensure preferential amplification of rare DNA targets. Another method commonly used in the study of predator–prey interactions is the design of species- or group-specific primers that target one or a group of species of interest [52]. Given that for the purpose of this study the species with abundant DNA is known (i.e. the captured teleost) and usually identifiable from its retrieved remains, and that the range of likely species responsible for depredation was at least partly known, the latter strategy was selected. The designed primers were demonstrated to be effective in selectively amplifying trace amounts of shark species DNA even in the presence of a vast excess of prey species DNA.

A range of other important assay design parameters must also be considered in any analytical method based on the identification of potentially forensic quantities of target DNA to ensure the generation of robust results that minimize the potential for both false positives and negatives. Trace samples are generally collected using multiple sterile swabs following identification of suitable target area [53]. We utilised a similar multiple swab approach, targeting the area of sample deposition associated with the actual bite.

While some loss of DNA is expected due to the influence of the substrate on which the sample is presented and the swabbing procedure adopted, a majority of DNA loss is considered to be due to the DNA extraction methodology [53]. We compared three commercially available extraction methodologies to ensure maximum retrieval of DNA. Amplification of trace DNA for all sixteen incidents proved that DNA was successfully extracted from the swabs. Another concern with the collection and analysis of trace forensic samples is the potential for the introduction of contamination during sample collection and laboratory processing. We incorporated a number of controls to detect any likely source of contamination and all controls performed as expected.

Understanding which species are responsible is key to development of mitigation strategies to reduce incidents of depredation. A recent review of shark depredation, [54] highlighted the lack of information available on the identification of depredating shark species. Identification of sharks responsible for depredation has relied on observations made by fishers of sharks caught in the process of consuming hooked fish [10] and stomach content analysis of sharks caught in the area [12]. Recently, underwater video cameras mounted on vertical longlines have been trialed by researchers to record depredation incidents [10]. The DNA based method, developed in this pilot study, provides a noninvasive and reliable approach to depredator identification as evidenced by the successful identification of shark species in the sixteen sampled incidents. This technique can be used to validate data recorded by video cameras. Molecular methods can increase the reliability and accuracy of depredator species identifications and could be particularly informative in sparsely studied, multi-predator systems [30]. Unambiguous identification provided by DNA analysis could verify when or if the responsible animal has been captured or killed; thus, DNA-based techniques could become a standard tool for use in wildlife attacks [27] and in incidences of depredation.

This study has shown that forensic quantities of shark DNA are transferred during predatory shark-fish interactions and has thereby demonstrated the potential application of the method to support the identification of shark species involved in interactions with humans and water sport equipment such as surfboards and kayaks. The methodology developed in this study provides a new, practical and effective tool that may be deployed in association with trained observers or, with appropriate experimental design, controls and training, citizens involved with science (e.g. recreational fishers) to provide improved data on species responsible for depredation incidents.

### Ethics statement

In Western Australia, the Animal Welfare Act 2002 does not require DPIRD to obtain a permit to use animals for scientific purposes unless the species are outside the provisions of the Fish Resources Management Act 1994 and Fish Resources Management Regulations 1995. Nonetheless, all sampling was undertaken in strict adherence to DPIRD Policy for the handling, use and care of marine fauna for research purposes. The study did not involve anesthesia, euthanasia, or any kind of animal sacrifice.
